# Potentially Low-Value Medical Interventions in Frail Older Adults Near the End of Life: A Narrative Review

**DOI:** 10.7759/cureus.114590

**Published:** 2026-08-16

**Authors:** Ayra Khan, Abdullah Khan

**Affiliations:** 1 Medicine, Independent Research, Surrey, GBR; 2 Paediatrics, St Peter's Hospital, London, GBR

**Keywords:** deprescribing, end-of-life care, frail elderly, low-value care, palliative care, polypharmacy, shared decision-making, treatment burden

## Abstract

Frail older adults approaching the end of life frequently receive multiple medications, diagnostic tests, hospital-based treatments, and procedures despite limited physiological reserve, shortened time-to-benefit, and changing goals of care. Low-value care in this setting is not defined solely by treatment cost or intensity. It refers to an intervention that is unlikely to provide meaningful benefit, has burdens that outweigh its expected benefit, offers benefit only beyond the patient’s anticipated survival, or conflicts with the patient’s values and priorities.

This narrative review examines low-value medical interventions in frail older adults near the end of life and considers the evidence supporting deprescribing, de-escalation, and goal-concordant care. The literature was organised into six domains: preventive medications, diagnostic testing and screening, hospital and intensive care interventions, nutrition and hydration, disease-directed treatment, and antimicrobial or symptom-directed therapy. Evidence is strongest for identifying potentially inappropriate preventive medications and reducing medication burden, although effects on survival, quality of life, hospitalisation, and function remain heterogeneous. Tools such as STOPPFrail can support structured medication review, but most deprescribing instruments have been developed largely from expert consensus and have limited clinical validation. Evidence concerning statin discontinuation suggests that stopping preventive statin therapy may be reasonable for selected patients with limited prognosis when cardiovascular benefit is unlikely to be realised within the patient’s remaining lifespan. Enteral tube feeding in severe dementia has not demonstrated clear survival or quality-of-life benefits, but available evidence is predominantly observational and affected by confounding. Intensive care, emergency hospitalisation, routine investigations, cancer screening, and invasive procedures may become of low value when they impose substantial burden without a realistic prospect of improving outcomes that matter to the patient. However, these interventions should not be discontinued solely because of age, frailty, dementia, or palliative care involvement. Decisions should incorporate prognosis, treatment indications, time to benefit, symptom burden, reversibility, risks of withdrawal, patient preferences, family perspectives, and available alternatives. The central clinical task is therefore not simply to provide fewer treatments, but to provide treatments that are proportionate, evidence-informed, symptom-responsive, and consistent with the older person’s goals. Future research should prioritise patient-reported outcomes, caregiver outcomes, equity, treatment-related harms, and implementation strategies across primary care, hospitals, nursing homes, and hospice services.

## Introduction and background

Population ageing has increased the number of older adults living with multimorbidity, frailty, dementia, and progressive organ failure. These conditions often coexist with complex medication regimens and repeated contact with healthcare services. As physiological reserve declines, the balance between the benefits and burdens of medical intervention becomes increasingly uncertain.

Frail older adults are particularly vulnerable to adverse drug events, functional decline, delirium, falls, treatment-related complications, and loss of independence. They are also frequently under-represented in clinical trials, meaning that evidence generated in younger or less co-morbid populations may not apply directly to people with advanced frailty. An age-blind approach may therefore lead to continued use of preventive treatments whose benefits require years to emerge, despite a much shorter life expectancy and a greater risk of harm [1].

Low-value care is commonly described as care that provides little or no benefit while exposing patients to potential harm, inconvenience, financial cost, or opportunity cost. In the context of end-of-life care, an intervention may be low value because it is physiologically burdensome, unlikely to change the outcome, inconsistent with the patient’s goals, or unlikely to provide benefit within the patient’s remaining lifespan. Low-value care should therefore be distinguished from care that is simply expensive, technologically intensive, or unsuccessful. An intervention may be appropriate when it offers meaningful symptom relief or supports a goal that the patient considers important.

Throughout this review, frailty, limited life expectancy, advanced dementia, hospice enrolment, and end-of-life status are treated as overlapping but non-identical clinical populations. Evidence derived from one population cannot automatically be generalised to the others. Accordingly, the population and clinical context associated with each major finding are identified in the relevant section, and evidence from advanced dementia, limited life expectancy, advanced cancer, or intensive-care populations is not assumed to apply to all frail older adults.

A systematic review of non-beneficial treatments in hospitalised patients near the end of life reported substantial use of resuscitation, intensive care, diagnostic testing, antimicrobial treatment, cardiovascular treatment, and other active interventions that were judged to offer limited benefit. The review also demonstrated wide variation in definitions and prevalence estimates, highlighting the difficulty of identifying low-value care using a single universal standard [2].

This narrative review examines the principal categories of potentially low-value medical intervention in frail older adults near the end of life. It focuses on the clinical evidence, ethical considerations, decision-making factors, and practical strategies that can support treatment proportionality and goal-concordant care.

Defining frailty and end of life

Frailty is a multidimensional state of increased vulnerability to stressors resulting from reduced physiological reserve. It may include physical weakness, slow gait, exhaustion, weight loss, cognitive impairment, dependence in activities of daily living, and reduced resilience after acute illness. Frailty should not be treated as synonymous with age. Chronological age, disability, multi-morbidity, dementia, and frailty overlap but represent different clinical constructs. 

The review included evidence involving frail older adults and related advanced-illness populations. Validated measures, such as the Clinical Frailty Scale [3], Fried frailty phenotype [4], Edmonton Frail Scale [5], and Frailty Index [6], were considered where reported, but the use of a validated frailty instrument was not an absolute inclusion criterion. The review also included studies defining eligibility through advanced dementia, limited life expectancy, advanced illness, hospice enrolment, or end-of-life status. These populations are overlapping but not interchangeable.

## Review

This narrative review used a structured, non-systematic approach to examine potentially low-value medical interventions in frail older adults near the end of life.

Search Strategy

A structured literature search was conducted across PubMed/MEDLINE, Embase, Scopus, Web of Science Core Collection, the Cochrane Library, and CINAHL using combinations of controlled vocabulary and free-text terms related to frailty, advanced illness, limited life expectancy, low-value care, treatment burden, de-prescribing, and the six predefined intervention domains. Searches were restricted to English-language publications.

The search strategy was organised into four conceptual blocks.

Population: Frailty, frail older adults, older adults, geriatric, Clinical Frailty Scale, Fried frailty phenotype, Edmonton Frail Scale, and Frailty Index.

Clinical context: Limited life expectancy, end of life, advanced illness, advanced dementia, hospice, palliative care, terminal illness, and serious illness.

Potentially low-value care: Low-value care, overuse, over-treatment, non-beneficial treatment, potentially inappropriate treatment, de-prescribing, polypharmacy, treatment de-escalation, treatment burden, and goal-concordant care.

Intervention: Preventive medications, statins, antihypertensives, diagnostic testing, screening, hospitalisation, intensive care, resuscitation, mechanical ventilation, dialysis, feeding tubes, enteral nutrition, artificial hydration, chemotherapy, disease-directed treatment, antimicrobial treatment, antibiotics, symptom-directed treatment, time-limited trials, and Patient Priorities Care.

Search structure:

(frailty OR frail older adults OR older adults OR geriatric OR Clinical Frailty Scale OR Fried frailty phenotype OR Edmonton Frail Scale OR Frailty Index) AND (limited life expectancy OR end of life OR advanced illness OR advanced dementia OR hospice OR palliative care OR terminal illness OR serious illness) AND (low-value care OR overuse OR overtreatment OR non-beneficial treatment OR potentially inappropriate treatment OR deprescribing OR polypharmacy OR treatment de-escalation OR treatment burden OR goal-concordant care) AND (preventive medications OR statins OR antihypertensives OR diagnostic testing OR screening OR hospitalisation OR intensive care OR resuscitation OR mechanical ventilation OR dialysis OR feeding tubes OR enteral nutrition OR artificial hydration OR chemotherapy OR disease-directed treatment OR antimicrobial treatment OR antibiotics OR symptom-directed treatment OR time-limited trials OR Patient Priorities Care).

Database-specific controlled vocabulary and syntax were adapted to the indexing system of each database rather than applying identical syntax across platforms. Searches were supplemented by backward and forward citation searching of key systematic reviews, clinical guidelines, consensus statements, and highly relevant primary studies. Particular attention was given to evidence concerning time to benefit, treatment burden, adverse outcomes, quality of life, patient and caregiver preferences, and goal-concordant decision-making.

Systematic reviews and meta-analyses, randomised clinical trials, cohort studies, observational studies, qualitative studies, clinical guidelines, consensus statements, and relevant narrative reviews were considered. Systematic reviews and clinical guidelines were prioritised when available, followed by randomised trials and high-quality observational studies. Qualitative studies, consensus tools, and narrative reviews were retained when they addressed patient or caregiver priorities, treatment burden, implementation, or areas in which comparative outcome evidence was limited.

Studies were excluded when they did not address the target population or clinical context; did not address one of the six predefined intervention domains; or did not provide information relevant to treatment benefit, harm, burden, appropriateness, patient preferences, or goal concordance. Paediatric studies, studies involving predominantly younger populations without separately reported older-adult data, case reports, small case series, conference abstracts without sufficient data, editorials, and commentaries without substantive evidence were excluded. Non-English publications were also excluded.

The review did not attempt to calculate pooled effect estimates. Instead, findings were synthesised thematically according to the expected benefit of the intervention, time to benefit, treatment burden, potential harms, patient preferences, and the degree to which the intervention was supported by clinical outcome evidence. 

Eligibility Criteria

Table 1 lists the eligibility criteria.

**Table 1:** Eligibility criteria

Eligibility Criteria for the Narrative Review		
Domain	Inclusion criteria	Exclusion criteria
Population	Adults aged ≥65 years; frail or prefrail older adults; populations identified using the Clinical Frailty Scale, Fried frailty phenotype, Frailty Index, Edmonton Frail Scale, Hospital Frailty Risk Score, or another validated frailty measure	Paediatric populations; predominantly younger populations; studies in which older or frail participants could not be separately identified
Clinical context	Limited life expectancy; advanced or serious illness; advanced dementia; terminal illness; hospice; palliative care; or care approaching the end of life	General healthy ageing or routine geriatric care without relevance to advanced illness, limited life expectancy, palliative care, or end-of-life decision-making
Interventions	Preventive medications; diagnostic testing and screening; hospitalisation; intensive care; CPR/resuscitation; mechanical ventilation; dialysis; artificial nutrition or hydration; feeding tubes; chemotherapy; other disease-directed treatment; antimicrobials/antibiotics; symptom-directed treatment; time-limited trials; priorities-aligned or goal-concordant care	Interventions unrelated to treatment intensity, treatment burden, de-escalation, appropriateness, or goal-concordant decision-making
Concept of interest	Potentially low-value care, overuse, overtreatment, non-beneficial treatment, potentially inappropriate treatment, deprescribing, polypharmacy, treatment de-escalation, treatment burden, or goal-concordant care	Studies addressing treatment efficacy alone without relevance to benefit, burden, harm, appropriateness, patient priorities, or treatment decision-making
Outcomes	Mortality; survival; functional outcomes; hospitalisation; ICU admission; treatment toxicity; adverse events; symptom relief; quality of life; treatment burden; healthcare utilisation; patient or caregiver preferences; goal concordance; decision-making outcomes	Studies reporting only outcomes unrelated to benefit, harm, burden, patient preferences, or clinical decision-making
Study designs	Systematic reviews and meta-analyses; randomised trials; cohort studies; observational studies; qualitative studies; clinical guidelines; consensus statements/criteria; relevant narrative reviews	Case reports; small case series; conference abstracts without sufficient data; editorials; letters without substantive evidence; commentaries without relevant evidence
Language	English-language publications	Non-English publications
Publication relevance	Studies informing one or more of the six predefined intervention domains	Studies with no substantive relevance to the review question or predefined intervention domains

Methods

The review included studies using validated measures, such as the Clinical Frailty Scale, Fried frailty phenotype, Edmonton Frail Scale, Frailty Index, or another clearly described instrument.

The term “near the end of life” also lacks a single operational definition. Studies may refer to the final months of life, an expected survival of less than 12 months, advanced progressive disease, severe dementia, hospice enrolment, or a clinical transition toward comfort-focused care. These categories are not interchangeable. Prognostic uncertainty is particularly substantial in frailty and dementia, where decline may be prolonged and punctuated by acute episodes.

For clinical decision-making, prognosis should be considered alongside functional status, treatment indication, time to benefit, treatment burden, and patient priorities. A limited life expectancy does not automatically make all preventive or disease-directed treatment inappropriate. Conversely, a patient does not need to be imminently dying for an intervention to become low value if its expected benefit is remote and its burden is immediate.

Preventive Medications and De-prescribing

Polypharmacy is common in older adults approaching the end of life. In a national cohort of more than 500,000 older adults who died in Sweden, exposure to 10 or more medications increased substantially during the final year of life. Preventive medications, including statins, antithrombotic agents, beta-blockers, and renin-angiotensin system inhibitors, continued to be used during the final month of life [7].

Evidence in this domain includes studies of frail older adults, people with limited life expectancy, people with life-limiting illness, and older adults approaching death. These populations are not identical, so findings concerning de-prescribing are interpreted according to the population and medication studied.

The clinical rationale for de-prescribing is strongest when a medication has a long time-to-benefit, limited current indication, substantial treatment burden, or potential to cause harm. Relevant harms include hypotension, falls, electrolyte disturbance, hypoglycaemia, bleeding, drug interactions, swallowing difficulty, confusion, and reduced adherence to symptom-relieving medicines.

STOPPFrail was developed to identify potentially inappropriate medications in frail older adults with limited life expectancy. The original consensus tool included criteria relating to lipid-lowering therapy, antihypertensive treatment, antiplatelet therapy, gastrointestinal medications, osteoporosis treatments, nutritional supplements, glucose-lowering therapy, and several other medication classes [8]. STOPPFrail version two further emphasised shared decision-making and included practical guidance for identifying patients approaching the end of life and reviewing antihypertensive, anti-anginal, and vitamin D therapy [9].

These tools should support, rather than replace, clinical judgement. A medication may be inappropriate for one patient but beneficial for another. For example, an antihypertensive agent may contribute to falls in a patient with orthostatic hypotension but reduce distressing symptoms in a patient with uncontrolled hypertension or heart failure. Similarly, an antiplatelet or anticoagulant may be preventive in one patient and essential for symptom or event prevention in another.

A systematic review identified 15 de-prescribing tools designed for frail older adults or people with limited life expectancy. Most tools were based on expert opinion, and only a minority had been evaluated in clinical practice [10]. This finding limits the certainty with which clinicians can predict the effects of de-prescribing on survival, function, hospitalisation, and quality of life.

The evidence supports a cautious but active approach. De-prescribing interventions generally reduce the number of medications or potentially inappropriate medications, but their effects on clinical outcomes are inconsistent. A systematic review of older adults with life-limiting illness found improvements in medication appropriateness, but concluded that evidence for quality of life, mortality, falls, and healthcare utilisation remained limited [11]. Another review of dual-purpose medications reported possible reductions in mortality and acute-care referral, but found insufficient evidence concerning quality of life, functional outcomes, hospitalisation, and adverse events [12].

Statins illustrate the importance of matching treatment to prognosis. A pragmatic randomised trial involving patients with an estimated life expectancy of one month to one year found no significant difference in 60-day mortality between patients who discontinued statins and those who continued therapy. Discontinuation was associated with slightly better quality-of-life scores, fewer medications, and lower medication costs [13]. These results support discussion of statin discontinuation in selected patients, but they should not be generalised to all older adults or to patients with recent acute coronary disease.

The statin discontinuation trial specifically involved patients with advanced, life-limiting illness, and an estimated life expectancy of one month to one year. Its findings support discussion of statin discontinuation in selected patients with limited prognosis, but they should not be extrapolated to all frail older adults or to patients with a strong recent cardiovascular indication.

A 2026 systematic review and meta-analysis of older adults with advanced frailty, dementia, or limited life expectancy found no statistically significant increase in mortality, hospitalisation, major adverse cardiovascular events, falls, fractures, or deterioration in quality of life following de-prescribing of selected preventive medications. However, the certainty of evidence was very low, heterogeneity was substantial, and the findings should not be interpreted as evidence that all preventive medications can be safely discontinued. Decisions should remain medication-specific and aligned with prognosis, indication, treatment burden, and patient priorities [14].

Diagnostic Testing and Screening

Diagnostic tests may become low value when the results are unlikely to change management or when subsequent investigations would impose greater burden than benefit. Examples include routine blood testing without a clear clinical question, repeated imaging for stable findings, monitoring of biomarkers that will not alter treatment, and cancer screening in patients whose life expectancy is unlikely to permit benefit.

Cancer screening requires particular attention to treatment preferences and expected harms. Patients with multi-morbidity and limited life expectancy may value screening for reassurance, information, or future planning, even when the likelihood of survival benefit is small. They may also underestimate the consequences of false-positive results, invasive follow-up procedures, overdiagnosis, and treatment complications [15].

The evidence discussed here primarily concerns patients with multi-morbidity or limited life expectancy rather than frailty defined by a validated instrument. The implications for frail older adults therefore depend on prognosis, functional status, treatment preferences, and whether the patient would pursue treatment following an abnormal result.

Shared decision-making should therefore explain both the potential benefit and the downstream pathway associated with a test. Screening should be reconsidered when the patient is unlikely to live long enough to benefit, would not pursue treatment for a detected condition, or would experience disproportionate burden from diagnostic follow-up. However, diagnostic testing remains appropriate when it may identify a reversible cause of distress, guide symptom treatment, or clarify a decision that matters to the patient.

Hospitalisation and Intensive Care

Hospitalisation may be appropriate when it provides treatment that is likely to improve comfort, function, or survival in accordance with the patient’s goals. It may become low value when it exposes a frail patient to delirium, immobility, falls, infection, loss of familiar surroundings, invasive procedures, or prolonged institutional care without a meaningful therapeutic objective.

Non-beneficial treatment at the end of life is not limited to intensive care. It may include repeated emergency department attendance, invasive monitoring, cardiopulmonary resuscitation, dialysis, transfusion, mechanical ventilation, or treatment escalation during irreversible deterioration [2].

Frailty is an important prognostic factor in intensive care. A systematic review and meta-analysis found that frailty was associated with higher hospital and long-term mortality and a lower likelihood of discharge home among patients admitted to intensive care [16]. These findings do not establish that intensive care is inappropriate for all frail patients. Instead, they support early assessment of baseline function, cognition, illness severity, reversibility, and the patient’s willingness to accept intensive treatments.

The available evidence applies to frail patients admitted to intensive care and demonstrates prognostic association rather than treatment futility. Higher mortality or lower likelihood of discharge home should not be interpreted as evidence that intensive care is inappropriate for every frail patient.​​​​​​​

Nutrition, Hydration, and Feeding Tubes

Eating and swallowing problems are common in advanced dementia and severe frailty. Families and clinicians may view feeding tubes as a means of preventing malnutrition, aspiration, pressure injuries, or death. However, the physiological decline associated with advanced dementia may not be reversed by artificial nutrition.

A Cochrane review of enteral tube feeding in severe dementia found no eligible randomised trials and included 14 controlled non-randomised studies. The available evidence was affected by substantial risks of confounding and selection bias, and it did not demonstrate a clear survival benefit [17]. The absence of high-quality evidence does not prove that tube feeding is never beneficial, but it limits claims that feeding tubes reliably improve survival or prevent complications. The evidence from this applies specifically to people with severe dementia and eating or swallowing difficulties.

Additional observational evidence has not demonstrated clear benefits of tube feeding over assisted hand feeding in severe dementia. In a retrospective cohort study, nasogastric feeding was not associated with lower rates of pneumonia, hospitalisation, or mortality than assisted hand feeding after adjustment for clinical factors. Similarly, a recent cohort study of patients with advanced dementia found that feeding-related hospitalisations were commonly associated with aspiration, while tube-fed patients experienced more complications at one year without a corresponding survival benefit. Both studies were observational and focused on severe or advanced dementia; their findings should therefore be interpreted cautiously and should not be automatically generalised to all frail older adults [18,19].

Feeding-tube decisions should distinguish nutrition as a medical intervention from eating as a social, cultural, and comfort-related activity. Careful hand feeding, assistance with preferred foods, treatment of dry mouth, modification of food texture, and attention to comfort may better reflect the goals of a patient who prioritises enjoyment, familiarity, and human contact.

Artificial hydration should likewise be considered individually, taking into account the patient’s symptoms, prognosis, route of administration, treatment burden, and goals of care. Although hydration may relieve selected symptoms, it may also contribute to oedema, respiratory secretions, breathlessness, repeated venous access, and monitoring burden [20].

In severe dementia, evidence regarding tube feeding outcomes beyond survival remains limited and predominantly observational. Available studies have not established consistent benefits for quality of life, nutritional status, aspiration-related complications, or pressure injuries, and the evidence is affected by substantial confounding and selection bias. Decisions should therefore be individualised, with careful hand feeding and comfort-focused care considered alongside the patient’s goals and preferences.​​​​​​​

Disease-Directed and Invasive Treatments

Disease-directed treatments can remain appropriate in palliative care when they relieve symptoms, prevent an imminent complication, or provide a benefit that the patient values. The transition to palliative care should not be interpreted as a requirement to stop all disease-directed treatment. Evidence in this domain is largely disease-specific and is not uniformly derived from studies of frail older adults.

Treatment may become of low value when the expected benefit is delayed, the treatment is unlikely to change the disease trajectory, toxicity is substantial, or the patient’s goals have shifted from life prolongation to comfort. Examples may include chemotherapy near death, repeated invasive cardiac procedures, renal replacement therapy without a goal-concordant benefit, or device therapy that prolongs dying without improving comfort.

The decision should include an explicit comparison between treatment continuation and treatment de-escalation. Clinicians should ask what the treatment is intended to achieve, how quickly benefit could occur, what burdens are expected, what would happen if treatment were stopped, and whether a less burdensome alternative exists.

​​​​​​​*Antimicrobial and Symptom-Directed Treatment*

Antimicrobials present a particular challenge because they may be used for cure, symptom relief, prevention of transmission, or life prolongation. Antibiotic treatment may be appropriate when it is likely to relieve distressing symptoms or treat a reversible infection that is consistent with the patient’s goals. It may be low value when it requires burdensome administration without a realistic prospect of symptom or outcome benefit.

Evidence concerning antimicrobial treatment near the end of life is heterogeneous and often comes from advanced cancer, terminal illness, dementia, or palliative-care populations rather than from frailty-defined cohorts. Findings regarding symptom relief, survival, adverse effects, and treatment burden should therefore be interpreted according to the underlying illness, infection, prognosis, and goals of care.

Antimicrobial treatment may remain appropriate when it is likely to relieve distressing symptoms, treat a reversible infection, or support a goal valued by the patient, but may become of low value when administration is burdensome, and there is little prospect of symptom or outcome benefit. Decisions should therefore distinguish symptom relief from infection cure, transmission prevention, and life prolongation. Disease-directed treatment should not be discontinued solely because a patient is receiving palliative care. It may remain appropriate when it offers symptom relief, prevents an imminent complication, or provides a meaningful benefit that the patient values; conversely, it may become disproportionate when benefit is delayed, toxicity is substantial, or treatment is unlikely to alter the disease trajectory or support the patient’s goals [21,22].

Symptom-directed treatment should not be categorised as low value merely because it does not prolong life. Opioids, anticonvulsants, anxiolytics, oxygen, diuretics, and other treatments may be central to comfort. The relevant question is whether the treatment improves an outcome that the patient values and whether its burdens are proportionate.​​​​​​​

Patient, Family, and Clinician Perspectives

Low-value care is influenced by more than clinical evidence. Patients and families may request intervention because they fear abandonment, believe that treatment represents hope, or misunderstand the likely consequences of escalation. Clinicians may continue treatment because of uncertainty, professional norms, fear of complaints, fragmented responsibility, or difficulty discussing prognosis.

Qualitative research suggests that older adults with limited life expectancy and their relatives may be open to de-prescribing when healthcare professionals initiate clear and respectful discussions. Participants often wanted information and involvement but did not necessarily know that medication reduction was an available option [23]. This evidence concerns older adults with limited life expectancy and their relatives and is used to inform communication and shared decision-making. It does not establish the clinical effectiveness of de-prescribing or treatment de-escalation in all frail older adults.

Communication should therefore frame treatment limitation as active care rather than abandonment. A conversation should include the patient’s understanding of the illness, their priorities, acceptable trade-offs, preferred place of care, concerns about hospitalisation, and views about treatments that may prolong life or increase burden.

Structured approaches, such as Patient Priorities Care, may help clinicians identify the outcomes that matter most to an older person and the treatments or burdens they are willing to accept. Such approaches may support medication review, referral decisions, hospitalisation planning, and discussions about life-sustaining treatment. However, they should complement rather than replace individual clinical assessment, prognosis estimation, and shared decision-making [24].

A time-limited trial may be useful when the outcome is uncertain. The clinical team can define the treatment goal, expected markers of improvement, duration of the trial, and circumstances in which treatment would be withdrawn. This approach may reduce conflict by replacing open-ended escalation with a transparent plan [25].

Shared decision-making is particularly important when the evidence is uncertain. The clinician should describe the best available evidence, explain uncertainty, identify the patient’s goals, and recommend a plan that is proportionate to the clinical situation. Family members should be involved according to the patient’s wishes and legal framework, particularly when cognitive impairment affects decision-making capacity.​​​​​​​

Equity and Ethical Considerations

Low-value care is not distributed equally. Differences in access to hospice, primary palliative care, specialist geriatric assessment, advance care planning, and culturally appropriate communication may influence whether a patient receives burdensome treatment. Conversely, efforts to reduce low-value care can become harmful if they result in undertreatment, discriminatory assumptions, or reduced access to potentially beneficial treatment.

Frailty, dementia, disability, and age should not be used as automatic reasons to deny treatment. Decisions should be individualised and based on expected benefit, treatment burden, prognosis, and patient preferences. The ethical objective is proportionality, not rationing based on age or disability.

Limitations

This narrative review has several limitations. The available literature uses inconsistent definitions of frailty, prognosis, treatment burden, and low-value care. Many studies are observational and vulnerable to confounding by indication, selection bias, and incomplete reporting of patient preferences. Frail older adults and people with advanced dementia are under-represented in randomised trials. Evidence from one healthcare system may not be transferable to another because treatment availability, reimbursement, cultural expectations, and palliative care access vary. Notably, evidence from one population should not automatically be generalised to frail older adults as a whole.

A further limitation is the use of evidence from overlapping but non-identical populations. Studies involving advanced dementia, limited life expectancy, advanced cancer, hospice enrolment, or intensive-care populations may provide relevant contextual evidence but cannot automatically be generalised to all frail older adults. The applicability of each finding depends on the population studied, the intervention indication, the prognosis, the outcome measured, and the patient’s goals of care. The narrative approach of this study permits integration of clinical, ethical, and qualitative evidence but does not provide the statistical certainty of a systematic review and meta-analysis.

This review has several strengths. It integrates clinical outcome evidence with qualitative, ethical, and implementation perspectives across six intervention domains. It also emphasises that potentially low-value care must be assessed in relation to prognosis, time to benefit, treatment burden, reversibility, and patient priorities rather than age, frailty, dementia, or palliative-care involvement alone. Finally, the review identifies important exceptions and areas in which evidence remains uncertain, thereby avoiding a blanket recommendation to withdraw treatment.

Research and medical education priorities

Future studies should use consistent definitions of frailty, limited life expectancy, low-value care, and treatment discontinuation. Research should report outcomes that matter to patients, including comfort, quality of life, function, cognition, caregiver burden, treatment regret, place of death, and continuity of care.

Randomised trials of de-prescribing and treatment de-escalation remain difficult but are needed where ethically and practically feasible. Observational studies should address confounding by indication and distinguish planned de-escalation from treatment cessation caused by clinical deterioration.

Studies should also examine how interventions affect different populations, including people with dementia, residents of long-term care facilities, people from minority ethnic groups, rural populations, and patients with limited access to palliative care. Implementation research is needed to determine how medication review, advance care planning, frailty assessment, and time-limited trials can be integrated into routine practice.

Research in this area is challenging because frail older adults near the end of life are heterogeneous in terms of frailty, dementia, multi-morbidity, prognosis, functional status, treatment indications, and personal goals. This makes it difficult to standardise study populations, interventions, and outcomes, while observational research remains vulnerable to confounding, selection bias, and incomplete documentation of patient preferences.

Future studies should report patient-centred outcomes such as comfort, quality of life, function, caregiver burden, treatment burden, and goal-concordant care. Medical education should also focus on frailty assessment, prognostic communication, shared decision-making, de-prescribing, time-limited trials, nutrition and hydration, antimicrobial use, and proportional treatment decisions. Implementation research is needed to integrate these skills across hospitals, primary care, nursing homes, and hospice services.

Table 2 lists the selected evidence informing potentially low-value care in frail older adults near the end of life, while Table 3 summarises this review.

**Table 2:** Selected evidence informing potentially low-value care in frail older adults near the end of life CPR, cardiopulmonary resuscitation; CFS, Clinical Frailty Scale; EOL, end of life; ICU, intensive care unit; PEG, percutaneous endoscopic gastrostomy; RR, risk ratio; OR, odds ratio; HR, hazard ratio

Domain	Study	Design	Population/Sample	Intervention/Exposure	Principal Findings
Frailty and end-of-life care	Stow et al. [26]	Systematic review and narrative synthesis	20 studies; 92,448 individuals, including 18,698 with frailty	End-of-life needs and preferences	People with frailty had substantial physical, psychosocial, and functional needs. Preferences for reduced intervention were reported but were not consistently reflected in care.
Preventive medications	Kutner et al. [13]	Randomised clinical trial	381 patients with advanced, life-limiting illness	Discontinuation versus continuation of statins	Statin discontinuation did not significantly worsen short-term mortality and was associated with modest improvements in quality of life and reduced medication burden and cost.
Preventive medications	Das et al. [14]	Systematic review and meta-analysis	15 studies; >33,000 participants	Deprescribing preventive medications	Deprescribing was not associated with increased all-cause mortality, hospitalisation, or major adverse cardiovascular events, although certainty of evidence was very low.
Preventive medications	Yourman et al. [27]	Meta-analysis	8 trials; 65,383 participants	Statins for primary prevention	Estimated time to benefit for prevention of one major adverse cardiovascular event per 100 treated adults was approximately 2.5 years.
Diagnostic testing/screening	Schoenborn et al. [28]	Longitudinal cohort	Older adults with limited life expectancy	Breast, colorectal, and prostate cancer screening	Cancer screening continued among some adults with limited life expectancy, with screening influenced by age, health, and functional status.
Diagnostic testing/screening	Smith et al. [29]	Systematic review	21 studies	Patient-reported cancer-screening decision-making	Age, health status, life expectancy, perceived cancer risk, screening beliefs, family influence, and clinician recommendations affected screening decisions.
Intensive care	Muscedere et al. [16]	Systematic review and meta-analysis	10 observational studies; 3,030 ICU patients	Frailty and ICU outcomes	Frailty was associated with higher hospital mortality, higher long-term mortality, and lower likelihood of discharge home.
Resuscitation	Hamlyn et al. [30]	Systematic review and meta-analysis	8 studies; 1,704 participants in meta-analysis	CPR in adults with frailty	Frailty was associated with substantially higher mortality following CPR (OR 3.56, 95% CI 2.74-4.63).
Nutrition	Lee et al. [31]	Systematic review and meta-analysis	12 studies/reports; 5,666 patients	Tube feeding in advanced dementia	Tube feeding was not associated with prolonged survival and was associated with increased mortality; sensitivity analyses suggested increased pneumonia and pressure sores.
Nutrition	Teno et al. [32]	Prospective cohort	36,492 nursing-home residents with advanced dementia and new eating problems	PEG feeding	PEG insertion was not associated with improved survival; timing of insertion was also not associated with survival.
Nutrition	Cai et al. [33]	Observational cohort	Nursing-home residents with advanced cognitive impairment	Hospital feeding-tube insertion patterns	Hospitals with more aggressive feeding-tube practices did not demonstrate improved survival.
Nutrition	Teno et al. [34]	Observational cohort	Nursing-home residents with advanced dementia	Feeding tubes and pressure-ulcer outcomes	Feeding-tube placement was not associated with prevention or improved healing of pressure ulcers.
Hydration	Bruno et al. [35]	Systematic review and meta-analysis	19 studies; 978 older adults	Hydration interventions	Evidence for interventions to improve hydration was heterogeneous, with limited evidence for consistent benefit across approaches.
Disease-directed treatment	Pearce et al. [36]	Systematic review and meta-analysis	58 studies	Frailty and systemic anticancer treatment	Frailty was associated with increased mortality, treatment toxicity, treatment intolerance, and hospitalisation.
Aggressive end-of-life treatment	Ma et al. [37]	Systematic review and meta-analysis	129 studies	Aggressive cancer care near end of life	Aggressive interventions, including late chemotherapy, ICU care, repeated hospitalisation, and emergency visits, remained common near death.
Antimicrobial therapy	Marra et al. [38]	Systematic review and meta-analysis	72 studies	Antibiotic use during end-of-life care	Forty-eight of 72 studies reported antibiotic use in >50% of patients; evidence concerning benefit and burden was heterogeneous.
Antimicrobial/symptom-directed therapy	Rosenberg et al. [39]	Systematic review	Hospice and palliative-care populations	Antimicrobials for symptom management	Evidence for symptomatic benefit was limited and heterogeneous, supporting individualised decisions.
Goal-concordant care	Tinetti et al. [40]	Nonrandomised clinical trial	366 adults ≥65 years with multiple chronic conditions	Patient Priorities Care	Priorities-aligned care was associated with greater reduction in treatment burden and more medications stopped, with fewer diagnostic tests and self-management tasks added.

**Table 3:** Summary of this study

Intervention domain	Representative potentially low-value interventions	Circumstances in which they may become low value	Important exceptions	Key burdens or harms	Evidence profile
Preventive medications	Long-term preventive statins, selected antihypertensives, glucose-lowering drugs, osteoporosis medications, antiplatelet or anticoagulant therapy	Long time-to-benefit, limited prognosis, weak current indication, immediate treatment burden, or increased risk of harm	Recent acute coronary disease, treatment of distressing symptoms, prevention of an imminent complication, or a clearly valued preventive goal	Falls, hypotension, bleeding, hypoglycaemia, electrolyte disturbance, drug interactions, swallowing burden, and reduced adherence to symptom-relieving medicines	Comparatively stronger evidence than in several other domains, but clinical outcomes remain heterogeneous. Deprescribing tools are largely based on expert consensus, while evidence for some individual drugs includes randomised and systematic-review data [7-14,27]
Diagnostic testing and screening	Routine blood tests, repeated imaging, biomarker monitoring, and cancer screening	Results are unlikely to change management, the patient would not pursue treatment, or the time-to-benefit exceeds expected survival	Investigation of reversible causes of distress, symptom-guiding tests, or tests needed for a decision that matters to the patient	False-positive results, overdiagnosis, invasive follow-up, anxiety, travel, financial cost, and treatment complications	Evidence is mainly contextual and observational, with limited direct evidence in frail older adults near the end of life. Shared decision-making and downstream consequences should be considered [15,28,29]
Hospitalisation and intensive care	Repeated emergency attendance, invasive monitoring, cardiopulmonary resuscitation, mechanical ventilation, dialysis, transfusion, and treatment escalation	Irreversible deterioration, no realistic therapeutic objective, or substantial burdens without expected improvement in comfort, function, or survival consistent with the patient’s goals	Reversible acute illness, meaningful symptom relief, or a patient-defined willingness to undergo intensive treatment	Delirium, immobility, falls, infection, invasive procedures, loss of familiar surroundings, and prolonged institutional care	Evidence includes a systematic review of non-beneficial treatments, a systematic review and meta-analysis of frailty in intensive care, observational research, and consensus or quality-improvement evidence concerning time-limited trials [2,16,25,30].
Nutrition, hydration, and feeding tubes	Feeding tubes and clinically assisted hydration in advanced dementia or severe frailty	Advanced irreversible decline, very limited prognosis, lack of expected benefit, or burdens that outweigh achievable goals	Reversible dysphagia, a temporary acute illness, a clearly defined treatment goal, or hydration for a specific distressing symptom	Tube-related complications, monitoring, repeated procedures, possible restraint, oedema, respiratory secretions, venous access, and loss of comfort-focused hand feeding	Tube-feeding evidence in severe dementia is predominantly non-randomised, with no eligible randomised trials in the cited Cochrane review and substantial risk of bias. Evidence for artificial hydration remains limited and highly individualised [17-20,31-35].
Disease-directed and invasive treatment	Chemotherapy near death, repeated invasive cardiac procedures, renal replacement therapy, and device therapy	Delayed benefit, substantial toxicity, little likelihood of changing the disease trajectory, or a shift toward comfort-focused goals	Symptom relief, prevention of an imminent complication, meaningful disease control, or a benefit explicitly valued by the patient	Toxicity, hospitalisation, procedural burden, complications, and possible prolongation of dying without improved comfort	Evidence is intervention- and disease-specific. It includes clinical trials, guidelines, palliative-care literature, and expert clinical judgement, but is not uniformly applicable to frail older adults [16,36,37].
Antimicrobial and symptom-directed treatment	Antibiotics or other antimicrobials when no meaningful symptom or outcome benefit is expected	Burdensome administration, no realistic prospect of symptom relief or cure, or treatment inconsistent with the patient’s goals	Reversible infection, expected symptom relief, prevention of transmission where relevant, or a patient preference for life prolongation	Intravenous access, adverse effects, drug interactions, monitoring burden, antimicrobial resistance, and possible hospital transfer	Evidence is heterogeneous and largely observational or consensus-based. Symptom-directed treatment should not be categorised as low value merely because it does not prolong life [21,38,39]

## Conclusions

Low-value medical intervention in frail older adults near the end of life is best understood through the relationship between expected benefit, time-to-benefit, treatment burden, prognosis, and personal goals. Preventive medications, routine testing, hospital-based escalation, feeding tubes, and invasive treatments should be reviewed regularly as illness progresses. These considerations must be applied within the population in which the evidence was generated because findings from advanced dementia, limited life expectancy, advanced cancer, or intensive-care populations should not automatically be extrapolated to frail older adults generally.

The appropriate goal is not to provide fewer treatments automatically. It is to provide care that is proportionate, equitable, compassionate, evidence-informed, and responsive to the older person’s values, symptoms, family circumstances, and preferred goals of care.
